# Multigenerational Rearing on Non-Prey Foods Does Not Affect Prey (Aphid) Recognition Behavior of *Coleomegilla maculata* (Coleoptera: Coccinellidae)

**DOI:** 10.3390/insects15110852

**Published:** 2024-10-31

**Authors:** Eric W. Riddick, Maria Guadalupe Rojas, Juan A. Morales-Ramos

**Affiliations:** Biological Control of Pests Research Unit, National Biological Control Laboratory, Agricultural Research Service, United States Department of Agriculture, Stoneville, MS 38776, USA

**Keywords:** biological control, rearing, *Coleomegilla*, herbivores, *Uroleucon*, *Aphis*

## Abstract

The pink spotted lady beetle *Coleomegilla maculata* has been identified as a promising predator to mass rear and release into greenhouses and high tunnels to control aphids on small fruits and vegetables. This study tested the hypothesis that laboratory-reared *C*. *maculata*, without any exposure to aphids for multiple generations, could recognize, attack, and consume live aphids. The aphid adults of two species were collected from non-crop host plants (weeds) over two consecutive seasons. The time (seconds) that *C*. *maculata* adults required to recognize and partially or completely consume live, healthy adult aphids was recorded in Petri dish arenas in the laboratory. Regardless of the non-prey food source (brine shrimp egg diet, mealworm-protein-based artificial diet), *C*. *maculata* adults readily recognized aphids. Adult females were occasionally more voracious than males. One aphid species (*Uroleucon erigeronense*) was consumed more readily than the other aphid species (*Aphis nerii*). In conclusion, multigenerational rearing on non-prey foods did not affect the prey recognition behavior of *C*. *maculata* adults in the laboratory. Validating the ability of lady beetles reared on artificial diets to recognize and consume live aphids is an important protocol before augmentative releases for aphid control in greenhouses and high tunnels.

## Simple Summary

This study evaluated the prey (aphid) recognition behavior of the pink spotted lady beetle (*Coleomegilla maculata*) adults that had been reared on non-prey foods for multiple generations. Regardless of the non-prey food source (brine shrimp egg diet, mealworm-protein-based artificial diet), predator adults readily attacked live aphids (two species) in Petri dish bioassays in the laboratory. Adult females were occasionally more voracious than males. One aphid species was consumed more readily than the other. In conclusion, the multigenerational rearing of pink spotted lady beetles on non-prey foods did not affect prey (aphid) recognition behavior in the laboratory.

## 1. Introduction

The pink spotted lady beetle *Coleomegilla maculata* DeGeer (*Cmac*) (Coleoptera: Coccinellidae) is distributed in North America as a native species with several subspecies found across the United States [1,2,3]. It is an effective predator of soft-bodied arthropods including aphids in numerous agroecosystems [4]. It can complete its development on plant pollen when prey are not readily abundant [5,6]. The ability to develop on plant pollen when prey species are scarce is an advantage to the mass rearing of *Cmac* in the absence of live prey.

*Cmac* has been mass-reared in laboratories for eventual release to control aphids and related pests as an alternative to using broad-spectrum insecticides in cropping systems. They are typically reared on live prey. Developing techniques to mass rear *Cmac* and other coccinellids on cost-effective foods such as artificial diets rather than live prey (aphids) is ongoing [7,8,9]. Moreover, developing protocols to evaluate the quality of mass-reared coccinellids is a critical need of the industry that produces natural biocontrol enemies [10,11,12,13]. In this study, the hypothesis that *Cmac* adults do not lose their capacity to recognize live aphid prey even after multigenerational rearing on non-prey foods was tested.

## 2. Materials and Methods

### 2.1. Coccinellid and Aphid Cultures

*Cmac* colonies have been in culture for more than a decade at the USDA, ARS, National Biological Control Laboratory (NBCL) in Stoneville, MS, USA. Information on the original individuals used to establish the colonies and the environmental conditions of rearing in climate-controlled rooms in the NBCL have been reported previously [14,15]. Aphids were collected from host plants in Stoneville during two consecutive seasons in summer and fall 2022 and 2023 just prior to experimentation. Two species were collected: *Uroleucon erigeronense* (Thomas) (Hemiptera: Aphididae) on horseweed *Erigeron canadensis* L. and *Aphis nerii* Fonscolombe (Hemiptera: Aphididae) on milkweed *Asclepias syriaca* L. Both aphid species were abundant in Stoneville. Aphid-infested foliage was carefully clipped (with scissors) from host plants each day and placed directly into clear plastic Ziploc^®^ freezer bags (S.C. Johnson Company, Racine, WI, USA) and immediately transported to the laboratory. Once in the laboratory, aphid-infested foliage was transferred to large clear plastic Petri dish arenas (14.5 cm diam, 2.5 cm height) lined with wax paper. Each arena had a screened lid for air circulation. The wax paper was lightly misted with distilled water prior to adding aphid-infested foliage. Next, arenas were stored in a laboratory refrigerator (at 10–12 °C, 45–50% RH) until experimentation.

### 2.2. Experimental Design and Treatments

Experiments were conducted on a laboratory benchtop under ambient conditions (22–23 °C, 31–34% RH, natural photoperiod). Individual aphids were isolated in medium-sized Petri dish arenas (2.5 cm high, 9.0 cm diam., 159 cm^3^) without screens fitted in the lids and exposed to *Cmac* adult attack, one adult per arena. Treatments consisted of *Cmac* deprivation (24 h vs. 48 h) and sex (males vs. females). Experiments were set up following a completely randomized design and tested the time (in seconds) for *Cmac* adults (males vs. females) to recognize prey, the time (in seconds) taken for adults to feed on prey, and the relative proportion of prey mass consumed by adults. *Cmac* adults reared on a factitious diet (BSE) or an artificial diet (AD) were challenged with adult aphids, *U*. *erigeronense* or *A*. *nerii*. BSE-reared and AD-reared adults were tested separately using the two aphid species in separate experiments. Stated briefly herein, the BSE diet consisted of brine shrimp *Artemia franciscana* Kellogg (Anostraca: Artemiidae) decapsulated egg powder plus green algae *Chlorella vulgaris* Beijerinck (Chlorellales: Chlorellaceae), and a fatty acid [16]. The AD diet was based on protein from *Tenebrio molitor* L. (Coleoptera: Tenebrionidae) pupal powder plus a mixture of other ingredients [17].

**Figure 1:**
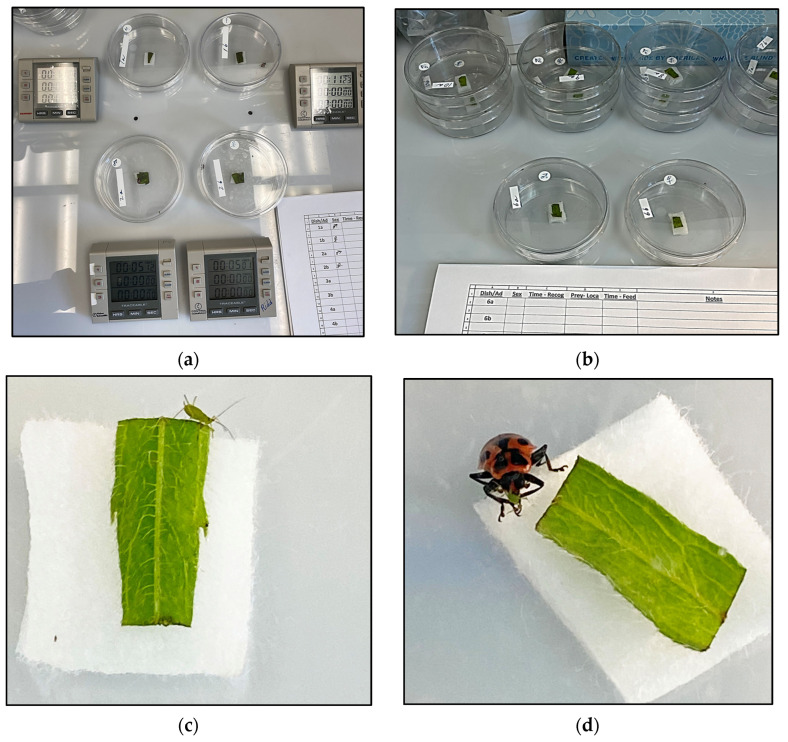
Images of experimental arenas with a water-soaked cotton platform topped with a piece of host plant foliage as the food source for aphids (**a**,**b**), closer view of aphid near edge of platform (**c**), and *Cmac* consuming aphid near edge of platform (**d**).

The BSE-reared colony and AD-reared colony have been in culture at the NBCL for more than 69 and 45 consecutive generations, respectively. The colonies have never crashed. The colonies have never been supplemented with individuals from wild (feral) populations. Background information on the aphids has been reported previously, indicating that *U*. *erigeronense* was consumed more readily than *A*. *nerii* [18,19,20,21,22,23]. The set up of the experimental arenas is illustrated in Figure 1a,b. The positioning of a water-soaked cotton platform topped with a piece of host plant foliage, inside the Petri dish, is illustrated. The foliage was a food source for aphids. Also, the foliage was from aphid-infested plants and presumably coated with aphid odors, i.e., chemical cues, attractive to *Cmac*. Note that both aphid species were mobile and often crawled off of the platform and onto the side wall, base, or lid of the Petri dish. An image of a *U*. *erigeronense* adult in an experimental Petri dish arena is provided (Figure 1c). The location of the aphid in the dish did not affect the time necessary for *Cmac* to attack, feed, or consume its prey. An image of *Cmac* consuming a *U*. *erigeronense* adult in an experimental Petri dish arena is provided (Figure 1d).

Experiments consisted of replicate trials testing BSE- or AD-reared *Cmac* males and females fed live, healthy adult aphids, *U*. *erigeronense* or *A*. *nerii* in 2022. Trial dates were 29–30 July, 3–4 August, and 5–6 August 2022 for BSE-reared *Cmac* offered *U*. *erigeronense* adults and 10–11 August and 12–13 August 2022 for BSE-reared *Cmac* offered *A*. *nerii* adults. Also, in 2022, trial dates were 24–25 August and 26–27 August 2022 for AD-reared *Cmac* offered *U*. *erigeronense* adults and 1–2 September and 7–8 September 2022 for AD-reared *Cmac* offered *A*. *nerii* adults. In summary, a total of 60 BSE-reared *Cmac* adults (30 males, 30 females) were tested against *U*. *erigeronense,* and 40 BSE-reared *Cmac* adults (20 males, 20 females) were tested against *A*. *nerii* in 2022. Also, a total of 40 AD-reared *Cmac* (20 males, 20 females) were tested against *U*. *erigeronense,* and 40 AD-reared *Cmac* (20 males, 20 females) were tested against *A*. *nerii* in 2022.

In 2023, replicate experiments tested BSE- or AD-reared *Cmac* offered *U*. *erigeronense* only. Trial dates were 1–2 June, 7–8 June, 15–16 June, 28–29 June, and 13–14 July 2023 for BSE-reared *Cmac* offered *U*. *erigeronense*. Trial dates were 24–25 May, 21–22 June, 19–20 July, and 24–25 August 2023 for AD-reared *Cmac* offered *U*. *erigeronense*. In summary, a total of 100 BSE -eared *Cmac* (50 males, 50 females) were tested against *U*. *erigeronense*. A total of 80 AD-reared *Cmac* (40 males, 40 females) were tested against *U*. *erigeronense*.

In all trials, *Cmac* adults (males and females) were approximately 20 days old and of the same generation when tested in the same experiment. There were 10 mating pairs (10 males, 10 females) involved in each replicate experiment. Mating pairs were separated into individual experimental arenas after 24 h or 48 h of food deprivation on the laboratory benchtop under ambient conditions (22–23 °C, 31–34% RH, natural photoperiod). Each adult was tested only once, then stored in the laboratory refrigerator. Adults were not returned to stock colonies.

### 2.3. Statistical Analysis

Initially, a two-factor analysis of variance (two-factor ANOVA) was used to test the influence of food deprivation and sex on the time it took *Cmac* adults to recognize, feed on, and consume prey. Since food deprivation was observed to have little or no effect on aphid recognition time or feeding time, and no adults died during the food deprivation period, these data were pooled. Likewise, data from experiments conducted in 2022 and 2023 (involving the same experimental design) were pooled to increase the robustness of the analysis. (Statistics generated from the two-factor ANOVAs of the 2022 and 2023 data can be made available upon request.) Student’s *t*-tests were used to determine sex effects on prey recognition time, feeding time, and proportion of prey body mass consumed in experimental arenas. Pearson product moment correlation analysis was used to determine if these data were correlated. Mean Student’s *t*-test values and correlation analysis statistic (*r*) values were considered significantly different if *p* < 0.05. A test for normality (Shapiro–Wilk), and a test for equal variance (Brown–Forsythe) was conducted prior to the two-factor ANOVA and Student’s *t*-test. SigmaStat^®^ interfaced through SigmaPlot^®^ for Windows V.15.0 (©2023, Systat Software Inc., San Jose, CA, USA) assisted with data analysis.

## 3. Results

### 3.1. Prey Recognition and Consumption Behavior of Cmac Adults Reared on BSE Diet

**Figure 2:**
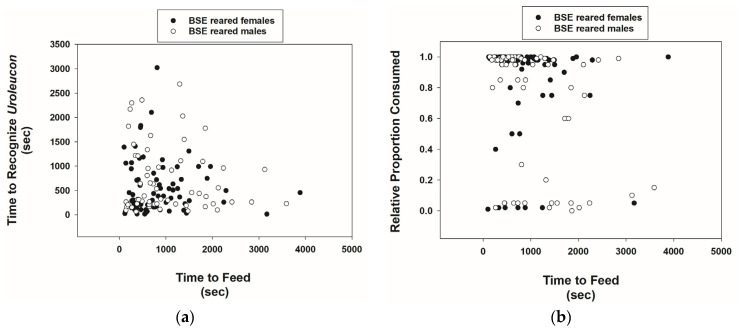
Scatterplots of the time (seconds) for *Cmac* adults reared on a BSE diet to recognize *Uroleucon erigeronense* adults vs. feeding time (**a**) and relative proportion of *U. erigeronense* adults consumed versus feeding time (**b**).

BSE-reared *Cmac* adults were highly capable of recognizing *U*. *erigeronense* adults. Out of a total sample size of 80 males and 80 females, only 7 males and 3 females failed to recognize prey. The time to recognize prey was highly variable, with a mean of 698.3 and 542.2 s for males and females, respectively (Table 1, Figure 2a). Females (rather than males) showed a tendency to take less time to recognize prey (Table 1). After prey recognition behavior, *Cmac* adults commenced feeding on prey for variable amounts of time. The time of feeding did not differ significantly between sexes. But, the relative proportion of prey consumed by *Cmac* did differ; females consumed more prey mass than males (Table 1). The mean ± SE proportion of prey consumed by *Cmac* females was 0.84 ± 0.03 (Table 1, Figure 2b). Note that there was a significant negative correlation between time of feeding and the proportion of prey consumed by males and females combined (*r* = –0.27, *p* = 0.001, *n* = 136).

**Figure 3:**
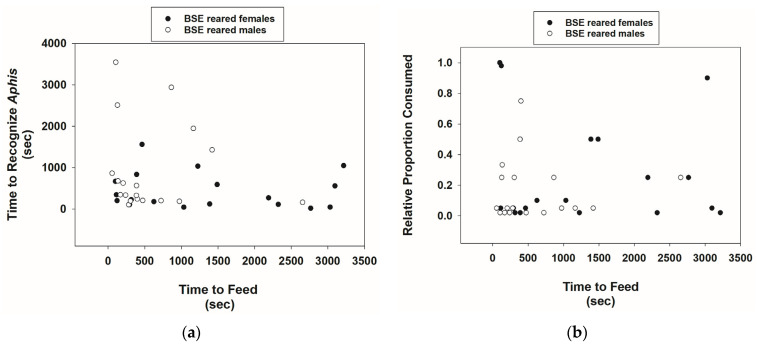
Scatterplots of the time (seconds) for *Cmac* adults reared on the BSE diet to recognize *Aphis nerii* adults vs. feeding time (**a**) and relative proportion of *A. nerii* adults consumed versus feeding time (**b**).

BSE-reared *Cmac* adults were also able to recognize *A*. *nerii* adults. Although sample sizes were smaller (20 males and 20 females) in this test than in the previous one, 0 males and only 2 females did not recognize prey. The time to recognize prey was variable, and there was a tendency for females to require less time to recognize prey (Figure 3a, Table 1). The time of feeding on prey was greater for *Cmac* females than males (Table 1). Yet, the proportion of prey mass consumed was considerably low for both males and females. The mean ± SE proportion of prey consumed by females was 0.27 ± 0.08 (Figure 3b, Table 1). There was no correlation between the time of feeding and the proportion of prey mass consumed by *Cmac* males and females combined (*r* = 0.013, *p* = 0.94, *n* = 37).

### 3.2. Prey Recognition and Consumption Behavior of Cmac Adults Reared on AD Diet

**Figure 4:**
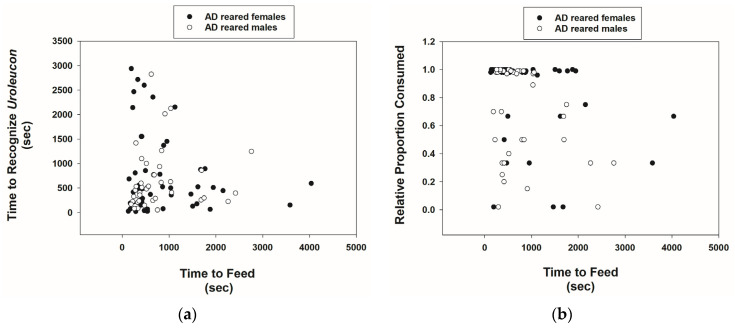
Scatterplots of the time (seconds) for *Cmac* adults reared on the AD diet to recognize *Uroleucon erigeronense* adults vs. feeding time (**a**) and relative proportion of *U. erigeronense* adults consumed versus feeding time (**b**).

AD-reared *Cmac* adults were also capable of recognizing *U*. *erigeronense* adults. Out of a sample size of 80 males and 80 females, just 8 males and 8 females failed to recognize prey. The time to recognize prey was variable (Figure 4a). Time of recognition did not differ significantly between males and females (Table 1). The time of feeding did not differ significantly between males and females, but females consumed more prey than males. The mean ± SE proportion of prey consumed by females was 0.86 ± 0.04 (Figure 4b, Table 1). Moreover, there was a significant negative correlation between feeding time and the proportion of prey mass consumed by males and females combined (*r* = −0.31, *p* = 0.002, *n* = 91).

**Figure 5:**
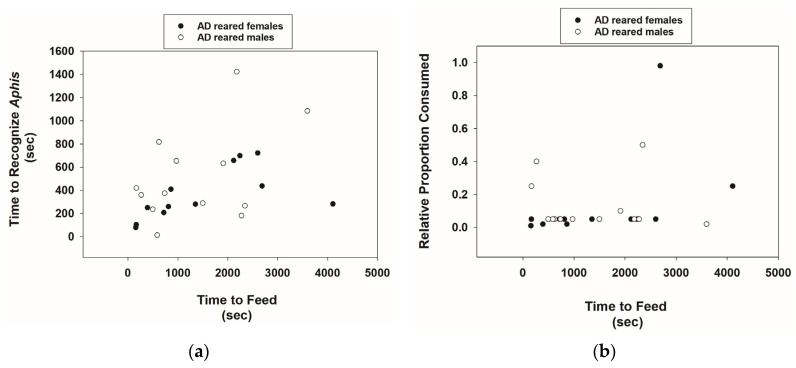
Scatterplots of the time (seconds) for *Cmac* adults reared on the AD diet to recognize *Aphis nerii* adults vs. feeding time (**a**) and relative proportion of *A. nerii* adults consumed versus feeding time (**b**).

AD-reared *Cmac* adults recognized *A*. *nerii* adults. The time to recognize prey was variable (Figure 5a), but recognition time did not differ significantly between males and females (Table 1). Note that there was a significant positive correlation between time to recognize prey and the time of feeding on prey (*r* = 0.46, *p* = 0.007, *n* = 33). The time of feeding on prey was also variable, but there were no significant differences between male and female feeding times (Table 1, Figure 5b). The proportion of prey mass consumed by *Cmac* males and females was low. For instance, the mean ± SE proportion of prey mass consumed by females was 0.11 ± 0.06 (Figure 5b, Table 1). The correlation between the time to feed and the proportion of prey mass consumed was not significant for males and females combined (*r* = 0.20, *p* = 0.26, *n* = 33).

## 4. Discussion

This study found that *Cmac* adults recognized and attacked live aphids, *U*. *erigeronense* and *A*. *nerii* adults. This observation was remarkable because of their lack of exposure to aphids, dead or alive. The BSE-reared and AD-reared colonies have been cultured on non-aphid foods for over 69 and 45 consecutive generations, respectively, in the NBCL. These colonies have never crashed, and wild (feral) adults have never been used to replenish either colony. The observation that the time to recognize prey was not affected by food deprivation (data not provided in the text) and only marginally by sex suggests that the rearing diets were nutritious. It could also suggest that the rearing diets did not obliterate the ability of *Cmac* adults to identify prey. Detection of live prey and readily feeding on aphids could represent an innate response not related to rearing history. More research on this topic is needed.

Another interesting observation was that *Cmac* females (rather than males) reared on BSE or on AD diets consumed relatively more mass of *U*. *erigeronense* than *A*. *nerii*. This observation was not completely unexpected. Firstly, *U*. *erigeronense* and other congeneric species have been considered as important prey to sustain coccinellid populations in landscapes surrounding agricultural fields [18]. Secondly, *A*. *nerii* have been found to contain toxic compounds, cardenolides, which negatively affected coccinellids [20,21,22,23]. Apparently, these compounds were distasteful to *Cmac* adults and hampered consumption potential in the experiments. Previous research noted that *A*. *nerii* was the least suitable prey of six aphid species, including *Uroleucon compositae* (Theobold), for the development and reproduction of the coccinellid *Coccinella septempunctata* L. [24]. Thirdly, there was a significantly negative correlation between feeding time and the proportion of *U*. *erigeronense* consumed by both the BSE-reared and AD-reared *Cmac* adults. Fourthly, *Cmac* females were typically larger than males and may have consumed more prey to satisfy nutritional requirements associated with egg development within their ovaries. The link between increased prey consumption and ovarian development in coccinellids has been confirmed in previous research [25].

In conclusion, the methods used in this study could be implemented by the biocontrol (natural enemy producer) industry to quickly test the quality of mass-reared predators, especially species that have undergone multigenerational rearing on artificial diets. Recording the time to recognize live prey for the first time of encounter, the time of feeding, and estimating the proportion of prey consumed after the first feeding bout could be incorporated into quality control guidelines. Establishing quality control guidelines is important to the biocontrol industry and for the future of the augmentative biological control of plant pests [9,10,11,12,13].

Questions regarding the perceived loss of prey recognition behavior in predators due to lack of contact after multiple generations were answered in this study. *Coleomegilla maculata* adults can readily identify and consume live prey even after multiple generations of rearing on an artificial diet. The use of artificial diets can reduce production costs by eliminating the need to rear prey (aphids).

## Figures and Tables

**Table 1:** Mean ± SEM time (seconds) necessary for BSE ^1^-reared or AD ^1^-reared *C*. *maculata* (*Cmac*) adults to recognize live prey for the first time, to feed on it, and the relative proportion consumed in laboratory arenas.

*Cmac* Diet	*Cmac* Sex	Prey	Time (s) to Recognize Prey	Time (s) of Feeding	Proportion of Prey Consumed
BSE	Male	*U*. *erigeronense*	698.33 ± 78.91 a	1017.10 ± 101.05 a	0.713 ± 0.050 a
	Female	*U*. *erigeronense*	542.17 ± 62.33 a	833.21 ± 78.35 a	0.843 ± 0.035 b
			*t* = 1.67	*t* = 1.44	*t* = 2.16
			*df =* 148	*df* = 134	*df* = 134
			*p* = 0.097	*p* = 0.15	*p* = 0.032
BSE	Male	*A. nerii*	900.35 ± 229.55 a	582.37 ± 145.32 a	0.183 ± 0.048 a
	Female	*A*. *nerii*	439.00 ± 103.18 a	1343.33 ± 269.17 b	0.271 ± 0.083 a
			*t* = 1.84	*t* = 2.42	*t* = 0.93
			*df* = 36	*df* = 35	*df* = 35
			*p* = 0.074	*p* = 0.021	*p* = 0.36
AD	Male	*U*. *erigeronense*	813.71 ± 128.618 a	770.77 ± 99.79 a	0.720 ± 0.052 a
	Female	*U*. *erigeronense*	727.83 ± 112.15 a	833.96 ± 114.25 a	0.862 ± 0.039 b
			*t* = 0.890	*t* = 0.154	*t* = 2.23
			*df* = 102	*df* = 89	*df* = 89
			*p* = 0.376	*p* = 0.878	*p* = 0.028
AD	Male	*A*. *nerii*	468.78 ± 83.19 a	1304.71 ± 239.44 a	0.131 ± 0.037 a
	Female	*A*. *nerii*	366.87 ± 56.15 a	1388.75 ± 296.73 a	0.111 ± 0.060 a
			*t* = 0.814	*t* = 0.047	*t* = 0.29
			*df* = 32	*df* = 31	*df* = 31
			*p* = 0.421	*p* = 0.963	*p* = 0.776

^1^ BSE, brine shrimp egg mixture; AD, mealworm-based artificial diet. See Section 2 for reference to *Cmac* diets. Mean values followed by a different letter in a column per experiment are significantly different (*p* < 0.05).

## Data Availability

The authors will consider placing data of reported results on ResearchGate (www.researchgate.net), if requested.
